# Degradation of Phenol via an Advanced Oxidation Process (AOP) with Immobilized Commercial Titanium Dioxide (TiO_2_) Photocatalysts

**DOI:** 10.3390/nano13071249

**Published:** 2023-03-31

**Authors:** Michael Schwarze, Steffen Borchardt, Marvin L. Frisch, Jason Collis, Carsten Walter, Prashanth W. Menezes, Peter Strasser, Matthias Driess, Minoo Tasbihi

**Affiliations:** 1 Department of Chemistry, Technische Universität Berlin, Straße des 17. Juni 124, 10623 Berlin, Germany; 2 Materials Chemistry Group for Thin Film Catalysis—CatLab, Helmholtz-Zentrum Berlin für Materialien und Energie, Albert-Einstein-Str. 15, 12489 Berlin, Germany

**Keywords:** phenol, AOP, TiO_2_, catalyst immobilization, TOC, cost estimation

## Abstract

Four commercial titanium dioxide (TiO_2_) photocatalysts, namely P25, P90, PC105, and PC500, were immobilized onto steel plates using a sol-gel binder and investigated for phenol degradation under 365 nm UV-LED irradiation. High-performance liquid chromatography (HPLC) and total organic carbon (TOC) analyses were performed to study the impact of three types of oxygen sources (air, dispersed synthetic air, and hydrogen peroxide) on the photocatalytic performance. The photocatalyst films were stable and there were significant differences in their performance. The best result was obtained with the P90/UV/H_2_O_2_ system with 100% degradation and about 70% mineralization within 3 h of irradiation. The operating conditions varied, showing that water quality is crucial for the performance. A wastewater treatment plant was developed based on the lab-scale results and water treatment costs were estimated for two cases of irradiation: UV-LED (about 600 EUR/m^3^) and sunlight (about 60 EUR/m^3^). The data show the high potential of immobilized photocatalysts for pollutant degradation under advanced oxidation process (AOP) conditions, but there is still a need for optimization to further reduce treatment costs.

## 1. Introduction

Phenol is a major pollutant in the industrial wastewater of the petrochemical industry that is harmful to the environment [1] and the European Union regulation no. 80/778/EC limits the maximum phenol concentration in drinking water to 0.5 mg L^−1^. The removal of phenol is, therefore, an important concern and various methods have already been summarized in a number of review articles [2,3,4]. The degradation of phenol via an advanced oxidation process (AOP) is a promising approach in which reactive oxygen species (ROS) are used to mineralize phenol to carbon dioxide (CO_2_) and water (H_2_O), instead of transferring phenol to another phase [5,6]. In addition to phenol degradation, AOP has been proven to be a powerful technique for micropollutant removal from aqueous solutions. Even though the ROS in the process can be released from dissolved precursors, e.g., hydrogen peroxide (H_2_O_2_), photocatalytic processes have gained considerable attention [7,8]. H_2_O_2_ is one of the most used ROS sources, but other precursors, such as sodium persulfate [9] or permonosulfate [10], can be used too. TiO_2_/UV AOP is quite common in contaminant degradation due to the abundance of TiO_2_ and its cheap price, non-toxicity, and photocatalytic stability [11]. However, TiO_2_ requires ultraviolet (UV) light owing to its large band-gap of about 3 eV to produce electron–hole pairs that are subsequently used to produce ROS from dissolved oxygen [11]. Even though, in most studies as well as in the present one, TiO_2_ is used in the form of simple particles, the photocatalytic performance can also be improved through the nanostructuring of TiO_2_ [12]. The TiO_2_/UV system has already been applied for the degradation of phenol [13,14,15] and other pollutants, such as dyes [16,17], pharmaceuticals [18,19], and plastics [20,21], by several researchers, with high degradation efficiencies. These very good efficiencies are often equated with an equally high mineralization, which is not necessarily the case. Photocatalytic degradation is still too seldom accompanied by the measurement of dissolved carbon content, which indicates the degree of mineralization. Several examples have shown that, although high-performance liquid chromatography (HPLC) or UV/Vis results indicate a high removal efficiency for the investigated pollutant, the degree of mineralization into CO_2_ and water is to a certain extent lower [22,23]. Since the residual carbon content plays a decisive role after photocatalytic treatment, the high degradation efficiencies often mean that treatment times are projected to be too short. This may change if TiO_2_ is immobilized for technical implementation. The immobilization of TiO_2_, for which different methods and supports have been published [24,25,26], avoids the issue of particle aggregation in a solution and allows the easy product (purified water) and catalyst separation after photocatalytic treatment. Therefore, the catalyst concentration can no longer be varied to the same extent as in the case with suspensions, and optimized catalyst loading has to be determined. In addition, the quality of the immobilization and the stability of the film play a decisive role in the technical implementation. In the context of optimizing the photocatalytic degradation process, one important question is hardly answered in the literature: what is the cost of the photocatalytic treatment of wastewater? Although the exact costs are likely to depend strongly on the system, an approximate order of magnitude would be very welcome for (a) the improvement of photocatalytic degradation processes and (b) comparison with other water purification technologies.

In this paper, the TiO_2_ modifications P25, P90, PC105, and PC500 were immobilized on steel plates and investigated for the degradation of phenol using a 365 nm UV-LED. Firstly, the impact of the oxygen source (air, dispersed synthetic air, and H_2_O_2_) was investigated to select an appropriate AOP system for further investigations. Secondly, the operating conditions for the most suitable AOP system were varied. Finally, a treatment plant was designed and the costs for water treatment based on the lab-scale results were calculated.

## 2. Materials and Methods

### 2.1. Chemicals and Photocatalysts

Phenol (99.5%, Roth, Karlsruhe, Germany) was used as the pollutant in the photocatalytic degradation tests. Synthetic air (20.5 ± 0.5 vol% O_2_, Air Liquide, Berlin, Germany) and hydrogen peroxide (H_2_O_2_, 30 wt%, Fisher Chemical, Schwerte, Germany) were used as the oxygen sources. For the immobilization of the photocatalysts onto the steel plates, a silica binder prepared from tetraethylorthosilicate (TEOS, 98%, Sigma-Aldrich, Schnelldorf, Germany), hydrochloric acid (HCl, 37%, Roth, Karlsruhe, Germany), 1-propanol (99.5%, Roth, Karlsruhe, Germany), 2-propanol (HPLC, VWR Chemicals, Dresden, Germany), and Levasil (Obermeier, Bad Berleburg, Germany) was used. As eluents in the HPLC analysis, acetonitrile (ACN, HPLC, VWR Chemicals, Dresden, Germany) and ultrapure water (Synergy UV system, Burlington, MA, USA) were used. As commercial titanium dioxide (TiO_2_) photocatalysts, P25 (99.5%, Evonik, Essen, Germany), P90 (100%, Evonik, Essen, Germany), PC105 (100%, Millenium/Cristal ACTivTM, Thann, France), and PC500 (100%, Millenium/Cristal ACTivTM, Thann, France) were investigated. All photocatalysts have been extensively characterized in our previous publications and the main characteristics are summarized in Table 1.

### 2.2. Immobilization of the TiO_2_ Photocatalyst Particles

The TiO_2_ photocatalyst particles were immobilized onto steel plates (3.4 cm × 3.4 cm, A = 11.56 cm^2^) using a sol-gel method with a silica binder [29]. For the preparation of the silica binder, about 1.7 mL of TEOS was mixed under stirring with 36 μL of HCl. Then, about 2.5 mL Levasil was added dropwise. Finally, about 7.8 mL of 2-propanol was added dropwise, and the solution was stirred overnight. For the immobilization of TiO_2_, 0.35 mL of the silica binder was mixed with 50 mg of the photocatalyst and 1.25 mL of 1-propanol. The mixture was stirred for about 8 min and further treated in an ultrasonic bath (Sonorex, Bandelin, Berlin, Germany) to obtain a homogeneous suspension. The plate was cleaned and roughened with an abrasive paper (P 1200, Hermes, Paris, France) before the photocatalyst immobilization. Layer after layer of the TiO_2_ suspension was applied to the plate with a brush. Before and after the application of each layer, the plate was placed in an oven (T = 60 °C) for about 30 min.

### 2.3. Degradation of Phenol

The degradation of phenol with the immobilized TiO_2_ photocatalyst particles was studied in a homemade crossflow photoreactor system (Figure S1 in Supplementary Materials). The prepared photocatalyst plate was placed in the main photoreactor, and the photoreactor was closed with a quartz glass window (5.3 cm × 5.3 cm × 0.3 cm). A total of 100 mL of the phenol solution (c_0,Phenol_ = 50 ppm) was added to a reservoir from where it was circulated (Ismatec pump, ISM597A) between the reservoir and the photoreactor at a flow rate of 0.2 mL s^−1^. Before the irradiation, the circulation was carried out under dark conditions for 30 min to obtain the adsorption–desorption equilibrium. Then, the circulation was continued under UV-LED irradiation (365 nm, 400 W m^−2^, Neumüller Elektronik GmbH, Weisendorf, Germany). The UV-LED was placed above the glass window (d = 1 cm). Samples (V_sample_ about 1 mL) were taken from the reservoir at different time intervals and analyzed via HPLC. The residual solution was also analyzed by TOC. During the advanced oxidation process, (a) hydrogen peroxide was added once to the reservoir or (b) the solution in the reservoir was permanently purged with synthetic air.

### 2.4. Data Evaluation

From the phenol concentration profiles, the degradation efficiency (DE) (also named as removal efficiency or removal rate) was calculated by using Equation (1) [30].

(1) $$\mathrm{DE}=\left(\frac{c_{0}-c_{t}}{c_{0}}\right)\cdot 100\%$$

In Equation (1), c_0_ is the initial phenol concentration and c_t_ is the phenol concentration after irradiation time t. The phenol adsorption during the dark phase is negligible (<5%). Equation (1) was also applied to calculate the degree of mineralization (DOM) using the carbon concentrations from TOC measurements. In addition, a pseudo-first-order (1st) kinetic model (Equation (2)) was used to determine the reaction rate constant (k_1st_) from the ln (c_t_/c_0_) vs. t plot [31].

(2) $$\frac{{\mathrm{dc}}_{\mathrm{Phenol}}}{\mathrm{dt}}=-k_{1\mathrm{st}}\cdot c_{\mathrm{Phenol}}$$

### 2.5. Analytic Methods

#### 2.5.1. HPLC

The phenol concentration in the liquid samples was measured via high-performance liquid chromatography (HPLC) using an Agilent 1200 chromatograph equipped with a Multospher 120 RP 18 (250 mm × 4 mm) column from Ziemer Chromatographie, Hamburg, Germany. As the eluent, a mixture of ACN and water (70:30) was used at a flow rate of 1 mL min^−1^, and the column temperature was 25 °C. Phenol and intermediates were detected at a wavelength of 225 nm. The phenol retention time was about 3.1 min. The samples were filtered through a cellulose acetate (CA) filter (13 nm, 0.2 mm, Membrane Solutions, Auburn, WA, USA). HPLC was calibrated with aqueous phenol solutions prepared from a phenol stock solution of 50 ppm.

#### 2.5.2. TOC

The carbon content in the samples was measured using a total organic carbon (TOC) analyzer (multi N/C 3100) from Analytic Jena, Jena, Germany. Because the required volume was large (ca. 20 mL), only the final sample was measured. The samples were acidified with 250 μL of 2N HCl, and they were purged with synthetic air using an appropriate TOC method to remove the dissolved CO_2_. For a standard 50 ppm phenol solution, the initial carbon concentration was calculated to be 38.3 mg L^−1^ by using Equation (3) (M is the molecular mass of carbon C or phenol).

(3) $$c_{0,C}=\frac{M_{C}}{M_{\mathrm{Phenol}}}\cdot c_{0,\mathrm{Phenol}}$$

#### 2.5.3. SEM

The morphology of the prepared photocatalyst films was investigated with scanning electron microscopy (SEM) using a Hitachi SEM type SU8030 microscope operated at an acceleration voltage of 10 kV and a probe current of 15 pA.

#### 2.5.4. SEM-EDX Mapping

Energy-dispersive X-ray (EDX) elemental mappings were acquired in a JEOL JSM-7401F SEM at 20 kV using a Quantax 400 (Bruker, Billerica, MA, USA) energy-dispersive X-ray spectrometer. The working distance was set to ~12.0 mm. At least three different areas were analyzed via EDX analysis for each sample. The measurement duration was set to at least 300 s. For the evaluation of the acquired spectra, the software Esprit (Bruker, V1.8.2 (2008)) was used.

#### 2.5.5. XRD

Powder and thin-film X-ray diffraction (XRD) measurements were conducted on Bruker D8 Advance instruments using Cu-K_α_ radiation (λ = 1.5409 Å, LynxEye, Karlsruhe, Germany detector). The diffraction patterns were collected in the range of 20–70° 2 θ angles (Bragg–Brentano geometry for the PXRD) with a step size of ~0.04°. Reflections were assigned using the PDFMaintEX library (v. 9.0.133).

## 3. Results and Discussion

### 3.1. Characterization of the TiO_2_ Films

**Figure 1:**
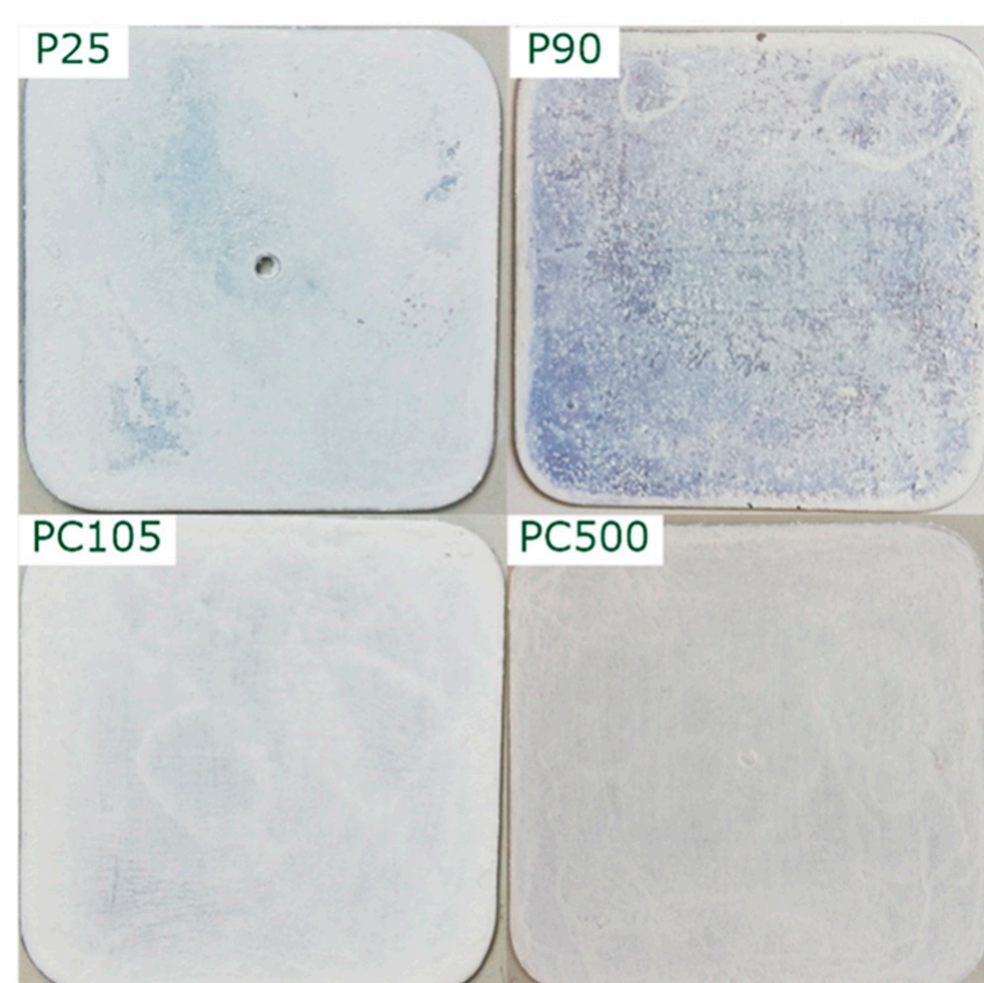
Supported TiO_2_ photocatalyst particles.

The four TiO_2_ modifications P25, P90, PC105, and PC500 were immobilized on steel plates using the sol-gel method (Figure 1). The amount of TiO_2_ was fixed at 50 mg so that the theoretical TiO_2_ loading of the plates was about 4.3 mg cm^−2^.

**Figure 2:**
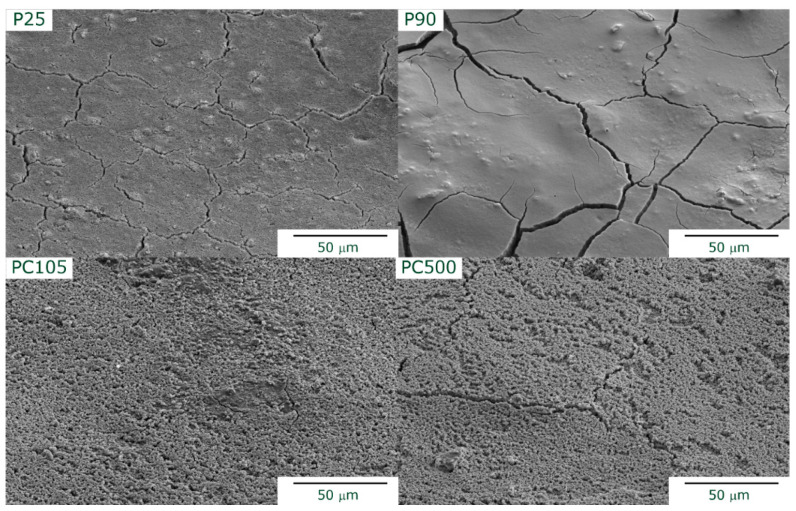
SEM images of the supported TiO_2_ photocatalysts (tilt angle = 50°).

It can be observed that the films are reasonably homogeneous, covering most of the underlying steel plate. An average film thickness of about (80 ± 20) μm was determined. For a better comparison of the films, SEM images were taken (Figure 2). P25 and P90 show compact surface films with macroscopic cracks, whereas PC105 and PC500 show a porous microstructure.

**Figure 3:**
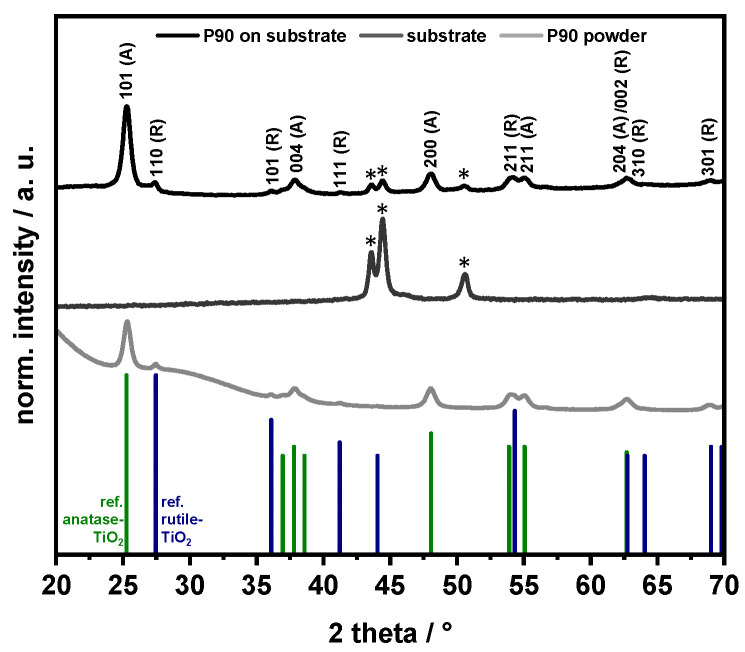
Results from the X-ray diffraction analyses in Bragg–Brentano geometry. Reference patterns for both anatase-TiO_2_ (JCPDS nr. 01-078-2486) as well as for rutile-TiO_2_ (00-021-1276) are provided as vertical bars in green and blue, respectively. Reflections indicated with an asterisk (*) belong to the steel substrate.

To ensure the successful immobilization of TiO_2_ onto the plates without changes to the material properties, XRD patterns for the P90 powder, the plate, and immobilized P90 photocatalyst particles were recorded (Figure 3).

**Figure 4:**
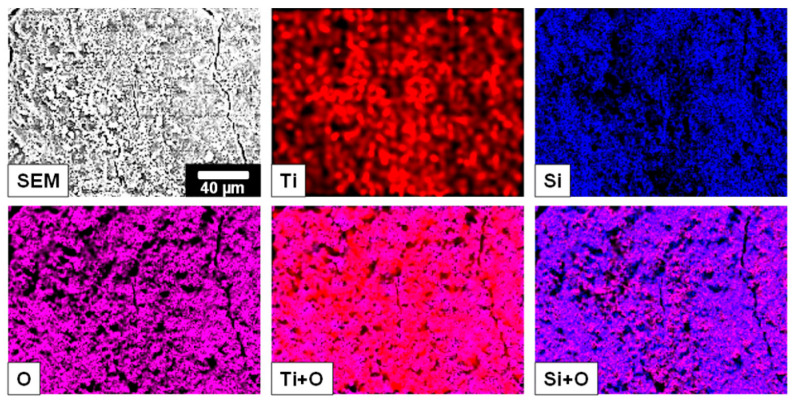
SEM-EDX elemental mappings of the immobilized P90 (the shown scale bar is valid for all images).

According to the PXRD pattern, the unsupported P90 powder shows a major and a minor oxide contribution, which can be assigned to polycrystalline anatase- and rutile-type TiO_2_, respectively. For the immobilized P90, both phases are still present with a similar ratio of the main reflections at ~25.3° (101) (anatase) and at ~27.5° (110) (rutile). In addition, weak reflections of the underlying steel support are found, as indicated by the asterisks (*) in Figure 3. The SEM-EDX elemental mappings confirmed a homogeneous distribution of the TiO_2_ particles over the entire plate, which is promoted by the presence of the silica binder (Figure 4).

After the preparation of the films, the supported photocatalysts were examined with respect to their mechanical stability in an ultrasonic bath. Within 30 min of treatment, there was a minor loss in weight of about 4%. Some TiO_2_ particles at the edges of the plates (Figure S2 in Supplementary Materials) were detached. The photocatalyst layer was thus stable and suitable for its use in the degradation of phenol.

### 3.2. Degradation of Phenol with Immobilized TiO_2_ Photocatalyst Particles

#### 3.2.1. System Selection

**Figure 5:**
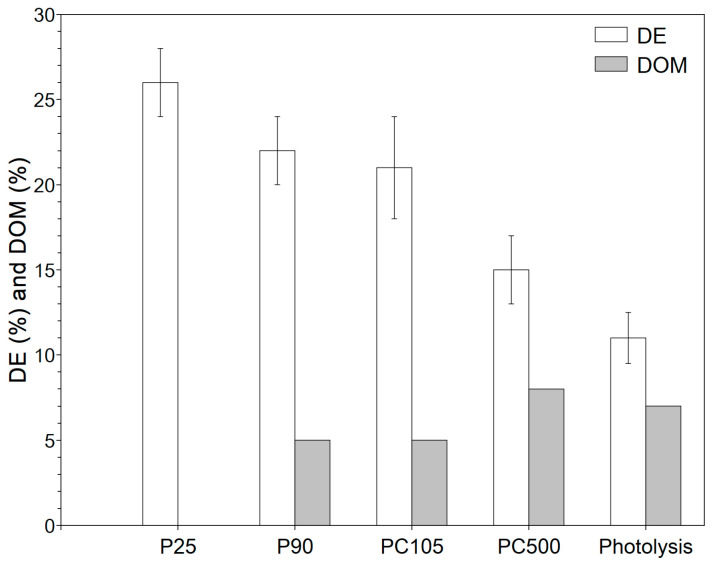
The degradation and mineralization of phenol (c_0,phenol_ = 50 mg L^−1^) using atmospheric oxygen after 3 h UV-LED (365 nm) irradiation with the supported TiO_2_ photocatalyst particles (4.3 mg cm^−2^) and without photocatalysts.

**Figure 6:**
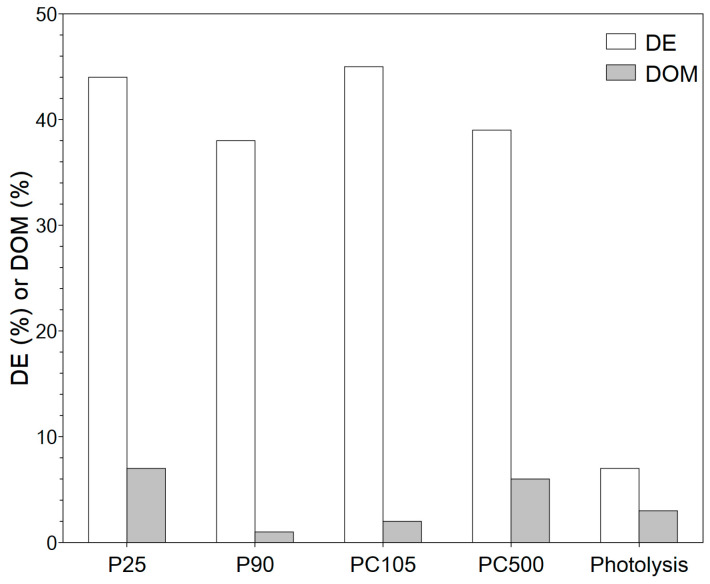
Degradation and mineralization of phenol (c_0,phenol_ = 50 mg L^−1^) using dispersed synthetic air after 3 h UV-LED (365 nm) irradiation with the supported TiO_2_ photocatalyst particles (4.3 mg cm^−2^) and without photocatalysts.

**Figure 7:**
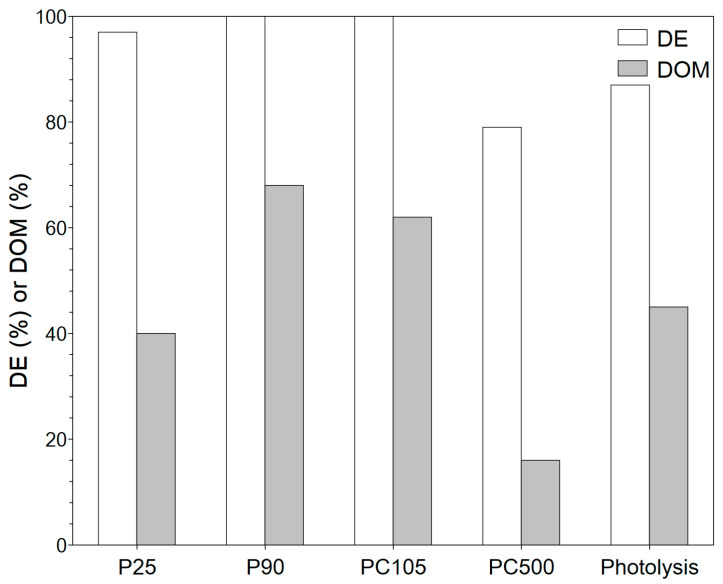
Degradation and mineralization of phenol (c_0,phenol_ = 50 mg L^−1^) using hydrogen peroxide (V_0,H2O2_ = 1 mL) after 3 h UV-LED (365 nm) irradiation with the supported TiO_2_ photocatalyst particles (4.3 mg cm^−2^) and without photocatalysts.

**Figure 8:**
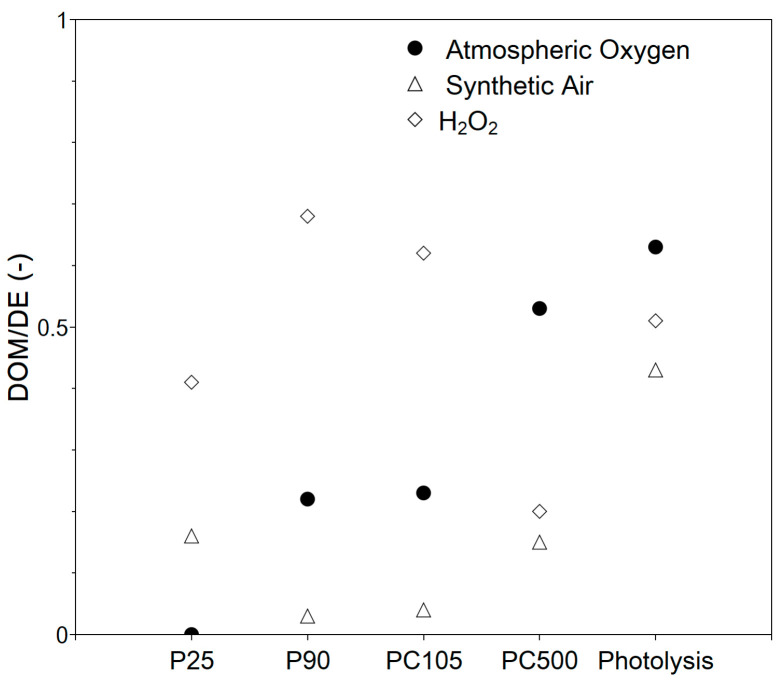
DOM/DE ratio for investigated degradation systems after 3 h UV LED (365 nm) irradiation with the supported TiO_2_ photocatalyst particles (4.3 mg cm^−2^) and without photocatalysts.

The successfully immobilized TiO_2_ photocatalyst particles were investigated for the degradation of phenol under AOP conditions. In this investigation, four commercial TiO_2_ photocatalysts were investigated that can be separated into two groups: pure anatase phase (PC105 and PC500) and mixed phase (P25 and P90). Furthermore, within each group, the photocatalysts differed in their surface area (P90 > P25 and PC500 >> PC105) and crystallite size (P25 > P90 and PC105 > PC500). The bandgap energy of all catalysts was the same (3.2–3.3 eV), which means that all TiO_2_ photocatalysts require UV light to be activated, but the differences in the observed activity were not related to light adsorption. The differences in surface area, crystallite size, and phase composition indicate that a direct comparison of the four photocatalysts is not possible. As the photocatalytic degradation of phenol under AOP conditions is strongly related to the oxygen source, the experiments were performed under atmospheric pressure, with dispersed synthetic air, and by adding H_2_O_2_. For each system, DE and DOM were determined (Figure 5, Figure 6 and Figure 7). For a better discussion and system selection for further investigations, the DOM/DE ratio was calculated (Figure 8) as an indicator for the relative quantity of mineralized phenol.

Evidently, both the way in which an oxygen source is offered to the system and which TiO_2_ modification is used have an impact on the degradation performance. The ideal case would be complete degradation (100% DE) with simultaneous complete mineralization (100% DOM), but for none of the systems studied could this be achieved within the 3 h irradiation time. Nevertheless, there were some differences between the systems, which are discussed in what follows. It is clear that the transformation of pollutants, in this case phenol, and mineralization into CO_2_ and water occur in several steps through intermediate stages, which has already been shown in the literature. This is also shown by the HPLC spectra taken at different time intervals (Figure S3 in Supplementary Materials). Trinh et al. postulated an intermediate phase where phenol transforms into other compounds, e.g., catechol or benzochinone, and a mineralization phase [32]. A possible degradation pathway for M/TiO_2_ (M = Cu, V, Cr) as a photocatalyst under UV irradiation, based on HPLC-MS results, is provided by Belekbir et al. [33]. The complex process of photocatalytic degradation focuses on the formation of reactive oxygen species (ROS), which are strongly influenced by the oxygen source and the properties of the catalyst. For this reason, three different systems for the formation of ROS were selected: atmospheric oxygen, dispersed synthetic air, and hydrogen peroxide. By comparing the immobilization and system, we could obtain suitable conditions for transfer to technology. First, the use of O_2_ from air or from a gas bottle with synthetic air is discussed. In the literature, many degradation experiments are carried out in an open stirred tank with irradiation from above using the oxygen dissolved under atmospheric pressure as the oxygen source. For atmospheric oxygen, DE is low with values from about 15% for PC500 to 25% for P25. The performance of PC500 is also worth being compared to that of P90, which is in agreement with our earlier investigations [34]. DOM values for the investigated TiO_2_ photocatalysts are below 10% and show an opposite trend with higher mineralization for PC500 compared to P25 (Figure 5).

Holm et al. studied the degradation of lactic and formic acid with commercial TiO_2_ photocatalysts without and with the addition of H_2_O_2_ [35]. They observed that mixed-phase catalysts are more active for degradation, and the activity of P90 was about 1.5 times higher than that of PC500. This result is in agreement with those of the present study. However, a higher activity is not a guarantee of better mineralization. The mineralization for PC500 is significantly better than that of P25. Lacheb et al. also obtained a similar result for the photocatalytic degradation of polynitrophenols using P25 and PC500 as the photocatalysts [36]. They argued that the higher surface area of PC500 is beneficial for the re-adsorption of the intermediates.

For oxygen from the atmosphere, active oxygen species, such superoxide radical ($O_{2}^{-˙}$, Equation (4)) and hydroxyl radical (${\mathrm{OH}}^{˙}$, Equation (5) or Equation (8)), are produced from dissolved oxygen and water in the presence of the photocatalyst [37].

(4) $$O_{2}+e^{-}\to O_{2}^{-˙}$$

(5) $$H_{2}O+h^{+}\to {\mathrm{OH}}^{˙}+H^{+}$$

(6) $$O_{2}^{-˙}+H^{+}\to {\mathrm{HOO}}^{˙}$$

(7) $$2{\mathrm{HOO}}^{˙}\to H_{2}O_{2}+O_{2}$$

(8) $$H_{2}O_{2}\to 2{\mathrm{OH}}^{˙}$$

When synthetic air is introduced into the solution with the aid of a gas frit, the formation of the ROS is favored because the aqueous solution is actively saturated with oxygen. The DE values significantly increase up to 40%, whereas the DOM values are still below 10% (Figure 6). A higher activity and lower mineralization are not contradictory, since more phenol molecules can be degraded in the first step in the same time window (in this paper, 3 h) due to a higher number of formed ROS, but the intermediates formed must react for mineralization to occur. As a result, the degradation rate constants (Table 2) increase. The constants are in the range of (0.5 to 1.4) × 10^−3^ min^−1^ and (0.3 to 3.0) × 10^−3^ min^−1^ for atmospheric oxygen and dispersed synthetic air, respectively. Compared to atmospheric oxygen, the constants are about two times higher. The rate constants in the presence of the catalyst are higher than those of photolysis, which shows that the catalyst is involved in the process. Compared to the literature, the rate constants are in the same order of magnitude [34]. As can be concluded from Figure 8, the use of dispersed synthetic air mainly favors fragmentation, as the DOM/DE ratio becomes lower. It might be easier to oxidize phenol rather than the intermediates. The experiments showed that the active gassing of the aqueous phenol solution is advantageous, as the intermediate phase is significantly accelerated. It has to be mentioned that no clear trend was observed for using dispersed synthetic air. The photocatalytic performance is a complex interplay of reactant/intermediate adsorption as well as the generation of the ROS. In the case of dispersed synthetic air, for the different photocatalysts, the type and number of formed ROS will be different. This step needs more investigation, which is not the focus of this work. Rather, with regard to a technical implementation, it should only be tested whether a significant increase in activity can be achieved through dispersion, possibly combined with better mineralization. As it can be seen, the activity can be significantly increased, but a better performance is obtained when H_2_O_2_ is added.

When H_2_O_2_ is used, the activation of dissolved atmospheric oxygen also takes place, but in addition, H_2_O_2_ mainly serves as the source of oxygen. The concentration of H_2_O_2_ has to be carefully selected as it has a double role in the degradation process. It can form more hydroxyl radicals (Equations (8) and (9)), but it can also act as an OH scavenger (Equation (10)) [37,38].

(9) $$H_{2}O_{2}+e^{-}\to {\mathrm{OH}}^{-}+{\mathrm{OH}}^{˙}$$

(10) $$H_{2}O_{2}+{\mathrm{OH}}^{˙}\to {\mathrm{HO}}_{2}^{˙}+H_{2}O$$

In the presence of H_2_O_2_, DE and DOM values increase considerably (Figure 7). Already in the absence of the photocatalyst, the degradation efficiency and degree of mineralization are about 90% and 50%, respectively. The H_2_O_2_/UV system is a very powerful AOP for pollutant degradation as shown in the literature [39,40,41]. Esplugas et al. showed that, within 30 min UV irradiation, up to 90% phenol degradation can be achieved depending on the H_2_O_2_ concentration, whereas in the absence of H_2_O_2_, the degradation efficiency is below 25% (depending on the pH value) [42]. A high OH radical concentration is directly obtained through the splitting of H_2_O_2_. The 365 nm UV-LED (UV-A) is able to split hydrogen peroxide into radicals, but compared to UV-C irradiation, which is favored in the H_2_O_2_/UV AOP, the number of hydroxyl radicals is lower. When the immobilized TiO_2_ photocatalyst particles are used together with H_2_O_2_, full degradation can be achieved and mineralization increases to almost 70% for P90. That the photocatalyst plays an important role in the TiO_2_/H_2_O_2_/UV system is obvious from PC500. PC500 shows the lowest DOM value with about 20%, even lower than the result of photolysis. As the concentration of H_2_O_2_ is kept constant, we would expect a similarly high DOM value if the catalyst plays only a minor role in the degradation of phenol using H_2_O_2_. The lower value indicates a lower concentration of ROS. When considering the phase composition of TiO_2_, it is shown that the anatase phase results in a lower radical formation rate compared to the mixed and rutile phases due to radical scavenging [43]. As PC500 consists only of the anatase phase, our result is in agreement with the literature. For P25, the DOM value is similar to that under photolysis, but for P90 and PC105, DOM values up to 70% are achieved under complete degradation, indicating that the combination of the photocatalyst and H_2_O_2_ can be beneficial, if it results in an increased formation of ROS. As P25 and P90 are mixed-phase TiO_2_ photocatalysts, their better performance compared to that of PC500 is also in agreement with the literature [43]. Experiments with the dispersed photocatalysts showed the same trend among the different modifications. Suspended PC500 particles show a low mineralization of approx. 36%, whereas 95% mineralization is achieved for the suspended P90 particles. The difference between the suspended and immobilized photocatalyst particles under otherwise identical conditions is due to the generally lower activity of the immobilized photocatalyst particles, since only a fraction participates in the reaction, and to the mass transfer limitations that can occur [44,45]. Therefore, more effort is required to achieve optimal film properties, e.g., layer thickness and surface area. The first-order-rate constant of the TiO_2_/H_2_O_2_/UV system is about one magnitude higher compared atmospheric oxygen and synthetic air, which is in agreement with the literature [15,42]. With respect to degradation and mineralization, the P90/H_2_O_2_/UV system was selected for further investigations. It should be noted that there are many studies in the literature on the use of different TiO_2_ modifications for the photocatalytic degradation of pollutants. The results depend on many factors, e.g., structural, optical, and electronic properties of TiO_2_ modification, the adsorption behavior of the pollutants and intermediates, and the experimental conditions. It is therefore not possible to make a general statement of which modification is better. It is reported that the same TiO_2_ photocatalysts P25, P90, and PC500 show an inverse order for the degradation of MB in an aqueous phase and the degradation of acetaldehyde in the gas phase [46] It seems that charge lifetime and surface area are the main parameters for activity. In several studies, P90 shows a very good activity, e.g., in the degradation of terephthalic acid (TPA) [47] or carbamazepine [48]. Surface potential (SPV) might be used to rank the TiO_2_ modifications with respect to charge separation efficiency/lifetime from which P25 should perform better in the aqueous phase [46] for phenol degradation. In the degradation of TPA, TiO_2_ films prepared via the sol-gel method were applied showing the better performance of the P90 film. From the experiments with different light intensities, the TiO_2_ layer had a better charge separation. This also indicates that, in the case of photocatalyst coatings, the film and film preparation might have an impact on the performance.

#### 3.2.2. Impact of Operating Conditions

Water quality: Chiou et al. [15] and other researchers have shown that the photocatalytic performance of phenol or pollutant degradation, in general, depends on a variety of experimental conditions, such as pollutant concentration and photocatalyst concentration, light source, and type and concentration of auxiliaries (e.g., H_2_O_2_ or co-catalysts). For immobilized catalysts, the type of support material and immobilization technique are also very important parameters. However, one of the most important parameters is the quality of the treated water stream. As an experiment on a lab-scale is usually carried out with distilled or pure water, the matrix effects of real wastewaters (such as ionic strength, pH, and other contaminants) are not considered. In order to obtain an impression of the loss in activity when changing the water quality, water from a local river was used as the solvent for phenol to simulate a real wastewater. The relative concentration profile of phenol, obtained from the HPLC results, is shown in Figure S4 in Supplementary Materials. The activity is lower compared to that of pure/distilled water, and only 60% degradation efficiency was observed. Due to the large carbon content in the real wastewater, TOC was not determined. This difference in photocatalytic activity was also observed by Jallouli et al., for the photocatalytic degradation of ibuprofen [18], or Kane et al., for the degradation of flumequine [49]. For a technical process based on photocatalysis, it is therefore very important to ensure a constant water quality. The question is whether photocatalysis should be used for all types of wastewater or only in very special cases where the composition is constant and known, e.g., in hospital wastewater. The laboratory tests show the maximum possible performance under defined reaction conditions, but the purification of contaminated aqueous systems to create the right environment for photocatalysis would be costly and time-consuming.

Light source: All experiments were performed with a 365 nm UV-LED (400 W m^−2^) because the activation of TiO_2_ requires wavelengths below 400 nm. To test whether wavelengths below 365 nm can improve the degradation of phenol, a UVC rod lamp with 254 nm (40 W m^−2^) was used. After 3 h of irradiation, DE was 100%, but the reaction was slightly faster for the UVC lamp with k_1st_ = 4.3 × 10^−3^ min^−1^. The DOM value for the UVC lamp was about 50% showing less mineralization compared to the UV-LED. The stronger UV light leads to a higher concentration of hydroxyl radicals that accelerates the fragmentation step. The better performance of the UVC lamp for phenol degradation, even at a lower intensity, was also shown by Morjène et al. [50]. However, the difference in the performance between the 365 nm UV-LED and the UVC lamp was low so that, for the TiO_2_/H_2_O_2_/UV system, 365 nm is already appropriate to achieve full degradation and good mineralization. This is an important aspect regarding the costs of water treatment if the degradation is carried out with artificial light sources rather than with sunlight.

Photocatalyst concentration: In photocatalytic experiments, a higher catalyst concentration might be beneficial for the degradation process when working with suspended photocatalysts. When the photocatalyst is immobilized on a substrate, only the photocatalyst particles in the upper layer of the immobilized film participate in the reaction, as the light is not able to reach the deeper layers. By increasing the catalyst concentration, only the film thickness increases, but the irradiation area is constant. The P90 concentration in the immobilization process was doubled, but the performance was the same as before, showing that the film obtained with 50 mg of P90 is already suitable to capture the photons for the activation of TiO_2_.

Flow rate and operation mode: The standard conditions for the investigation of phenol degradation with the immobilized P90 photocatalyst were 0.2 mL s^−1^ and the circulation of the phenol solution. With an initial volume of 100 mL, the entire liquid was circulated 22 times within the 3 h of irradiation. When the flow rate was lowered to 0.1 mL s^−1^, which corresponds to 11 liquid cycles, the reaction rate was lowered (k_1st_ = 2.2 × 10^−3^ min^−1^), but after 3 h of irradiation, the DE (98%) and DOM (65%) values were almost the same. The photocatalytic performance might be increased further for higher flow rates, which could not be established in our setup. The better photocatalytic performance is due to an increased turbulence in the system and mass transfer from the bulk solution to the surface of the immobilized P90 photocatalyst, as also mentioned by Argurio et al. [49]. However, the flow velocity must be selected so that the immobilized catalyst film remains stable. In the photocatalytic setup, the liquid was always circulated between the reservoir and the photoreactor, which is the operation mode of a closed-loop reactor. Alternatively, the phenol solution was not circulated and the liquid was collected in a separate vessel after passing the immobilized P90 photocatalyst. To enable a higher residence time in the full continuously operating photoreactor, the flow rate was lowered to 0.04 mL s^−1^ and, additionally, the dark period was skipped. As expected, the DE (49%) and DOM (13%) values were lower. In comparison to the standard experiment, the time under irradiation is too short. A better performance might be achieved with an even lower flow rate or a longer photoreactor.

### 3.3. Plant Design and Cost Estimation

**Figure 9:**
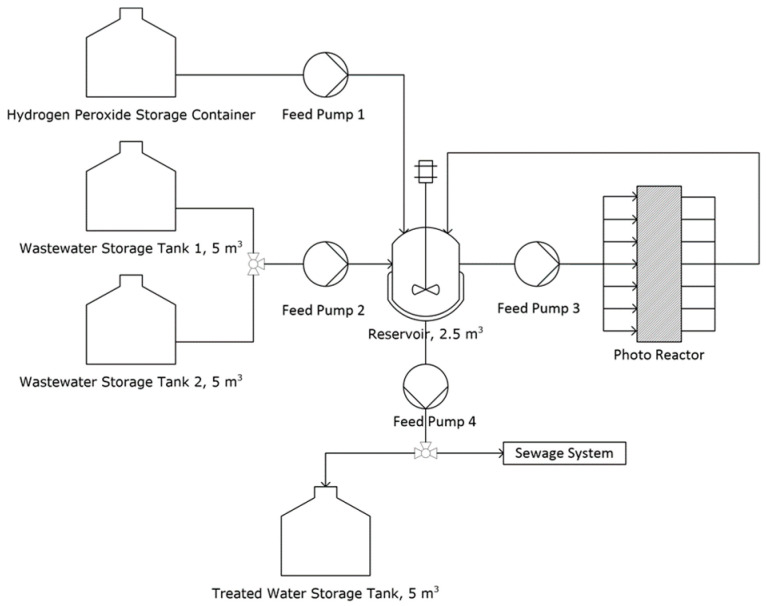
Concept for a phenol-containing wastewater degradation unit.

Based on the laboratory results with the immobilized P90 photocatalyst and hydrogen peroxide, a suggestion for a phenol-containing wastewater treatment plant (Figure 9) was made, and the treatment costs were estimated.

In this concept, the wastewater is first collected in a storage tank and then pumped together with hydrogen peroxide to a reservoir. From there, it is pumped into the photoreactor and back again until the appropriate degree of degradation/mineralization is reached. The treated water can then either be collected for internal reuse or discharged into an external water system (e.g., sewer system). There are different approaches to cost estimation and, in this case, the factorial method described in the book of Sinnot and Towler was used [51]. More details about the cost estimation are provided in the Supplementary Materials. As AOP will play a much larger role in wastewater treatment in the future, the main objective is to develop a sense for the treatment costs and to assess the overall potential of this method. The method presented in this paper is certainly not calculated down to the smallest detail, but it provides a very good impression of the order of magnitude of the treatment costs. When estimating the costs, the main focus is on the equipment and energy costs, which usually account for the largest share of the costs. With the exception of the photoreactor, the costs for the equipment shown in the process diagram (Figure 9) can be easily estimated. For the photoreactor, there is still no general model on which to build. For this reason, a photoreactor was first designed having the characteristics of the laboratory reactor, but being able to treat the amount of 2.5 m^3^.

Even though there are different types of photoreactors published in the literature [52,53,54], they have one thing in common: a large irradiation size. This is beneficial for the direct capture of sunlight. For a better utilization and higher light intensity, solar collectors can be used. To design the large-scale photoreactor, a simple scale-up in size was performed. The estimated prototype had an irradiation area of about 27 m^2^, where the photocatalyst was immobilized onto 25 plates (each 1.00 m × 1.08 m). The large area was also due to the fact that only a small film of liquid is allowed above the catalyst plate in order to avoid strong light attenuation. This size might be too large to be applied in a real wastewater treatment plant, but it allows for cost calculation. Furthermore, the reactor was aimed to work under real sunlight. If the plant operates with an artificial light source, other reactor geometries and constructions can be considered, such as a stack reactor with alternating plates and light sources. So, the reactor could be built with height rather than area in mind.

In addition to the cost of the photoreactor and other equipment, the cost of the irradiation itself is of particular importance. Of importance is also whether the irradiation is carried out with an artificial light source, as in the laboratory scale, which is available at any time of the day, or whether the irradiation is carried out with sunlight, which limits the time of operation considerably. Both lead to different costs for the irradiation and thus to the costs of the treatment. A summary is provided in Table 3. It should be mentioned that the costs of sunlight irradiation were calculated considering a similar photocatalytic performance as that of the 365 nm UV-LED. Morjène et al. [50] investigated the impact of the light source in the absence of H_2_O_2_, and the performance of a solar simulator was about half the performance of a UVC lamp, which was close to our 365 nm UV-LED. Therefore, the assumption is quite justified, and similar results should be obtained within 3 h of irradiation with sunlight.

Evidently, the treatment costs are much higher when using the UV-LED because of both the high number of required LEDs and the required electrical power. By simply scaling-up in size to retain the characteristics of the laboratory reactor, a large number of LEDs is required to be installed on the calculated area of 27 m^2^. This design seems very strange at first sight, but its technical implementation would be possible, although perhaps it is not the design of choice. As already mentioned, the construction should also be able to work directly with sunlight without any further modifications, which is why a planar surface was chosen. For the irradiation, the number of LEDs was therefore adjusted, which leads to a large investment and thus to greater costs. It can be assumed that this construction also deliberately leads to higher treatment costs and the achieved value already represents an upper limit for the treatment with an artificial light source. As an alternative to the LEDs, UV tubes could be considered, but these have a shorter lifetime and are not as flexible to install as LEDs. As already mentioned, other reactor concepts could also be considered for the technical implementation of the P90/H_2_O_2_/UV, if the UV light is to be generated by an artificial light source. A photoreactor that works on the principle of immersion lamps and can irradiate a large internal surface area would be a promising alternative to the planar reactor. However, the use of sunlight would only be possible to a limited extent. In cost estimation, sensitivity analyses are used to evaluate the influence of individual factors on the total costs. It was assumed that a different design would lead to 50% of the original cost of the artificial light source, but the remaining costs would not change significantly. This results in treatment costs of about 300 EUR/m^3^. The costs for sunlight are lower by a factor of ten and might already be within an acceptable range. The costs of conventional water treatment depend on the kind of pollutants to be removed and the used method. Joss et al. [55] mentioned the estimated costs for micropollutant removal via ozonation and activated carbon filtration of 0.05 EUR/m^−3^ and 0.20 EUR/m^−3^, respectively. For pollutants that cannot be removed in this way, or if the pollutant load is too high, water incineration is an established process. This is significantly more energy-intensive, even though some of the energy used can be recovered. Even if the costs for photocatalytic treatment are currently still high, they provide an order of magnitude and show whether there is potential for optimization. The costs can be reduced by producing more photocatalytic reactors, but certainly better photocatalysts that work very efficiently with sunlight have to be developed first in order to avoid the electricity and investment costs for artificial light sources.

## 4. Conclusions

Four commercial TiO_2_ modifications (P25, P90, PC105, and PC500) were immobilized on steel plates using the sol-gel method. The obtained photocatalyst films were homogeneous and showed good mechanical stability. The films were investigated for the photocatalytic degradation of phenol as a model pollutant, focusing on degradation and mineralization depending on the oxygen source (air, synthetic air, and hydrogen peroxide). Within 3 h irradiation with a 365 nm UV-LED under circulation (0.2 mL s^−1^) of the phenol wastewater, the P90/UV/H_2_O_2_ system showed the best performance with 100% degradation and 70% mineralization. Different operating parameters were investigated (such as catalyst concentration, light source, and flowrate), and showed the dependencies known from the literature. However, water quality is certainly of decisive importance with regard to technical realization. The photocatalytic treatment should be used if the water composition is not too complex, since the activity is significantly reduced (almost half in this work) by the accompanying substances. Based on the results for P90/UV/H_2_O_2_, a treatment plant was designed and the treatment costs were estimated for (a) sunlight and (b) 365 nm UV-LED as the light sources. For the UV-LED, due to the required electric current, the cost was about 600 EUR/m^−3^, but it might be further reduced using other irradiation concepts. Assuming that a similar performance can be achieved with sunlight, the cost was lower by a factor of 10, but it is at present still higher than the cost of standard processes, such as ozonation or filtration with activated carbon. The cost of UV-LEDs is similar to that of water incineration and can certainly be further reduced if (a) the treatment plant is optimized, (b) several plants of the same type are built, (c) more effective photocatalysts for the visible spectrum are developed, and (d) the optimal film properties are adjusted. The results clearly show that photocatalytic wastewater treatment on a technical scale is feasible with immobilized catalyst films.

## Figures and Tables

**Table 1:** Crystallite size (CS), BET surface area (SA), anatase (A) and rutile (R) phase composition, and band gap energy (BGE) of the investigated TiO_2_ photocatalysts.

Photocatalyst	CS (nm)	SA (m^2^ g^−1^)	A:R	BGE (eV)	References
P25	18.1	56	82:18	3.2	[27]
P90	12.6	102	87:13	3.3	[27]
PC105	20.9	80	100:0	3.3	[28]
PC500	6.0	270	100:0	3.3	[28]

**Table 2:** First-order reaction rate constant after 3 h UV-LED (365 nm) for the degradation of phenol (c_0,phenol_ = 50 mg L^−1^) with the supported TiO_2_ photocatalyst particles (4.3 mg cm^−2^) with atmospheric oxygen (AtmO), synthetic air (SynA), and hydrogen peroxide (V_0,H2O2_ = 1 mL).

Photocatalyst	k_1st, AtmO_ (10^−3^ min^−1^)	k_1st, SynA_ (10^−3^ min^−1^)	k_1st, H2O2_ (10^−3^ min^−1^)
Photolysis	0.5	0.3	10.6
P25	1.7	3.0	19.0
P90	1.4	2.3	33.9
PC105	1.3	2.7	29.1
PC500	0.9	2.2	7.8

**Table 3:** Results of the cost estimation for the phenol wastewater treatment plant (WWTP) with sunlight irradiation and the 365 nm UV-LED (OpEx = operational expenditure; CapEx = capital expenditure; V = volume).

	With Sunlight	With LED
V_day_ (m^3^)	5	5
V_Year_ (m^3^)	1150	1150
OpEx (EUR/Y)	4130	191,600
CapEx (EUR)	656,710	4,657,086
Treatment costs (EUR/m^3^)	61	572
Total treatment costs (EUR/Y)	70,150	657,800

## Data Availability

Data available on request.

## Supplementary Materials

The following supporting information can be downloaded at: https://www.mdpi.com/article/10.3390/nano13071249/s1, Figure S1: Scheme of the laboratory photoreactor (A) as well as photographs of the photoreactor with the immobilized TiO_2_ photocatalyst (B) and 365 nm UV-LED (C); Figure S2: Immobilized TiO_2_ photocatalyst before (A) and after (B) the mechanical stability test; Figure S3: HPLC chromatograms for phenol degradation using dispersed synthetic air (top) and hydrogen peroxide (down); Figure S4: Phenol degradation profiles for the simulated and real wastewaters; Figure S5: Scheme for cost estimation; Figure S6: Scheme of the WWTP photoreactor; Table S1: Values for the factorial method; Table S2: C_e_ values (in Euro) for the individual components for Germany in 2022; Table S3: Parameters for the laboratory and WWTP reactors; Table S4: Individual contributions to ISBL; Table S5: Calculation of CapEx; Table S6: Calculation of OpEx; Table S7: Calculation of the total treatment costs. References [51,56] are cited in the Supplementary Materials.
