# Supplementary material for: Degradation of Phenol via an Advanced Oxidation Process (AOP) with Immobilized Commercial Titanium Dioxide (TiO2) Photocatalysts
Source: Nanomaterials (Basel). 2023 Mar 31;13(7):1249. doi: 10.3390/nano13071249 (PMC10097325; doi:10.3390/nano13071249)
Supplement: Supplementary file 1 [file nanomaterials-13-01249-s001.zip › nanomaterials-2296853-supplementary.pdf]

nanomaterials

Supplementary Materials

Degradation of Phenol via an Advanced Oxidation Process (AOP) with Immobilized Commercial Titanium Dioxide (TiO_2) Photocatalysts

Michael Schwarze [1,*], Steffen Borchardt [1], Marvin L. Frisch [1], Jason Collis [1], Carsten Walter [1], Prashanth W. Menezes [1,2], Peter Strasser [1], Matthias Driess [1] and Minoo Tasbihi [1,*]

[1] Department of Chemistry, Technische Universität Berlin, Straße des 17. Juni 124, 10623 Berlin, Germany
[2] Materials Chemistry Group for Thin Film Catalysis—CatLab, Helmholtz-Zentrum Berlin für Materialien und Energie, Albert-Einstein-Str. 15, 12489 Berlin, Germany
* Correspondence: ms@chem.tu-berlin.de (M.S.); minoo.tasbihi@tu-berlin.de (M.T.)

1. Photocatalytic Setup

The photocatalytic degradation tests were conducted in a homemade photocatalytic setup consisting of a planar photoreactor with a slot for the immobilized photocatalyst, a reservoir for the phenol solution, a pump for liquid circulation, and a 365 nm UV-LED as the light source. The setup is shown in Figure S1.

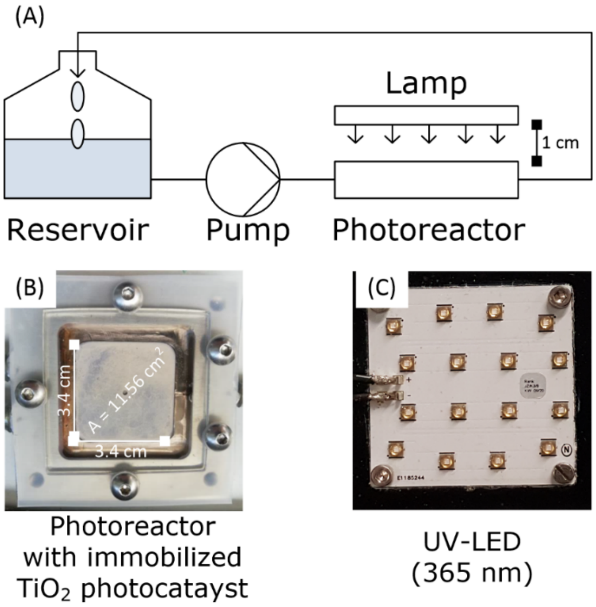

Figure S1. Scheme of the laboratory photoreactor (**A**) as well as photographs of the photoreactor with the immobilized TiO_2 photocatalyst (**B**) and 365 nm UV-LED (**C**).

2. Stability of the Supported Photocatalyst

The immobilized TiO_2 photocatalysts were treated in an ultrasonic bath to prove their mechanical stability. The mass lost was negligible and only at the edges of the immobilized film, some photocatalyst peeled off. The TiO_2 film before and after the treatment is shown in Figure S2.

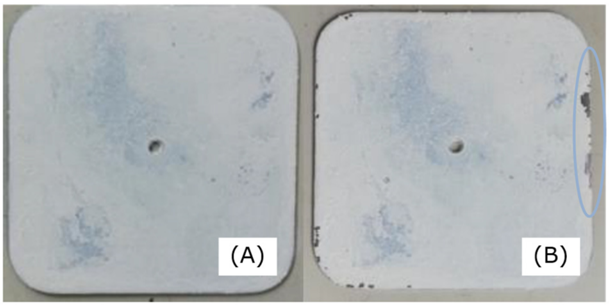

Figure S2. Immobilized TiO_2 photocatalyst before (**A**) and after (**B**) the mechanical stability test. Blue circle indicates loss of catalyst during mechanical stability test.

3. HPLC Analysis of the Phenol Solutions

The degradation of phenol was studied using liquid samples, which were analyzed by HPLC and TOC analyses. The HPLC spectra at the beginning and after 45 min of UV-LED irradiation using synthetic air or hydrogen peroxide are shown in Figure S3. It is obvious that the degradation proceeds over several intermediates.

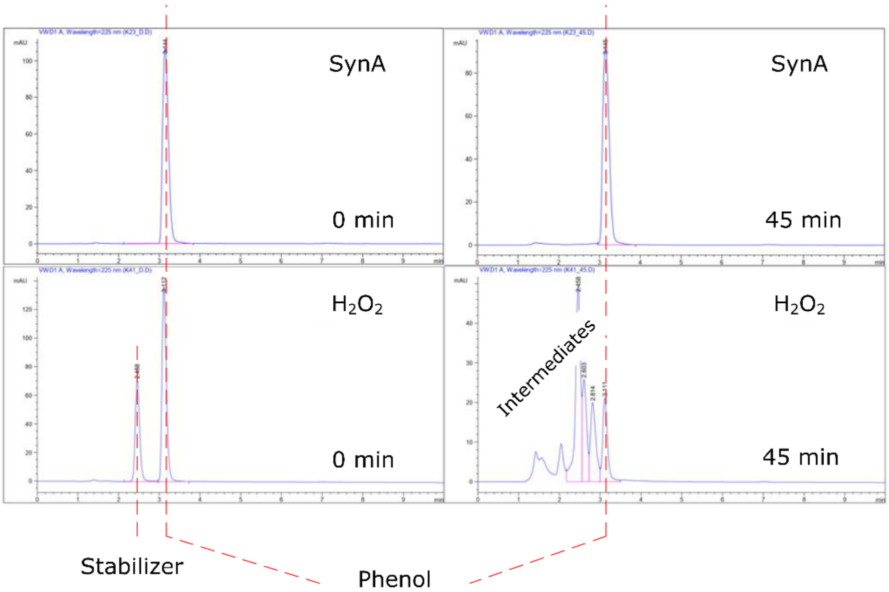

Figure S3. HPLC chromatograms for phenol degradation using dispersed synthetic air (top) and hydrogen peroxide (down).

4. Optimization

The impact of real water on the photocatalytic performance of the immobilized P90/H_2O_2/UV system is shown in Figure S4.

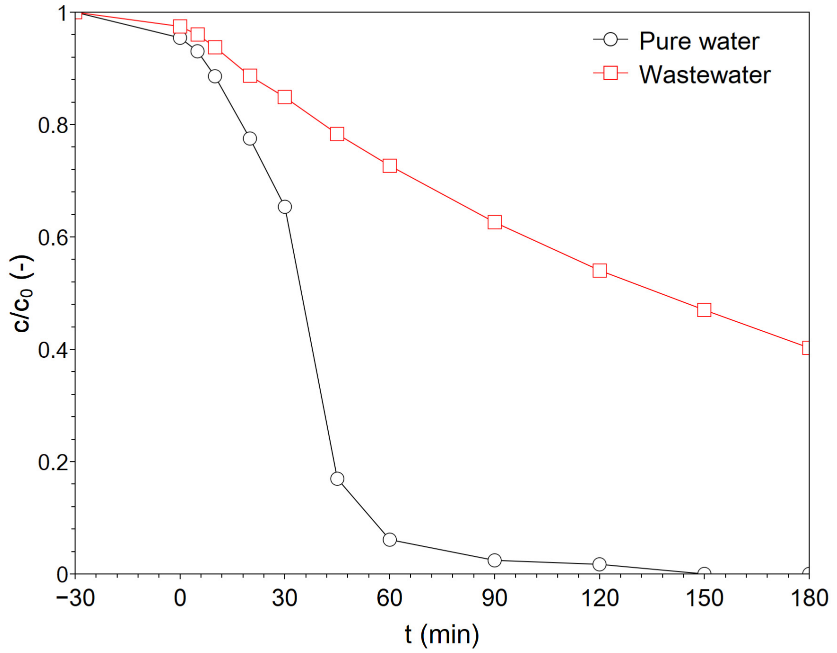

Figure S4. Phenol degradation profiles for pure water and wastewater.

5. Cost Estimation

In order to estimate whether a process is profitable, the cost with which a product can be offered on the market (COGS) must be estimated. The cost structure is shown in Figure S5 and consists of various parts, where the manufacturing costs (COGM) are of primary importance. The actual estimation of the costs of the plant components occurs within the framework of Capital Expenditures (CapEx) and, therefore, this was further subdivided. CapEx consists of Fixed Capital Investment (FCI), Working Capital (WC), and Starting Expenses and Contingency. The FCI is usually calculated in detail and the other two costs are determined by factors depending on the FCI. The FCI can be divided into Outside Battery Limit Costs (OSBL) and Inside Battery Limit Costs (ISBL). The ISBL consists of direct costs, which include the components that are actually installed in the plant and their cost to build, as well as indirect costs, which consist of the cost of engineering, construction supervision, and construction of the plant, including insurance. The direct costs are once again divided into equipment costs and other costs. Equipment costs are the costs of, for example, reactors, pumps, and heat exchangers, and other direct costs include piping, electrical, and plant control units. OSBL includes all costs for infrastructure to be created to operate the plant.

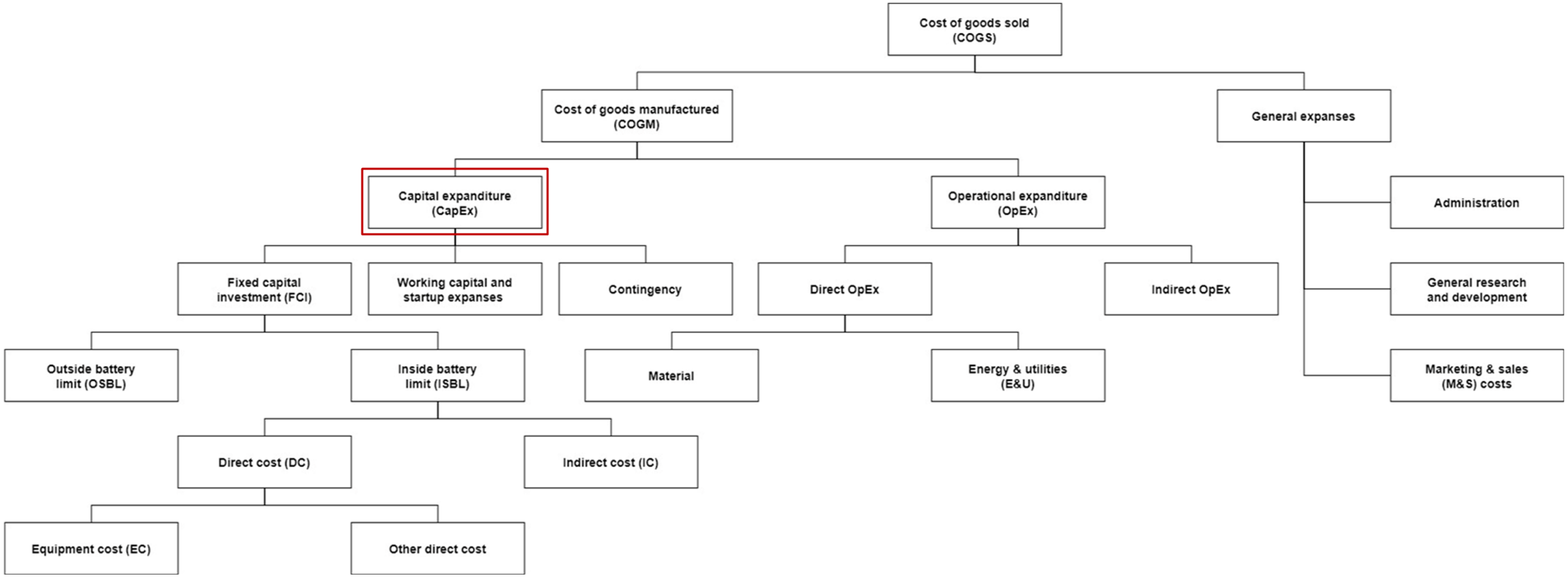

Figure S5. Scheme for cost estimation.

A detailed factor method was used to calculate the ISBL, according to which the costs of the ISBL are as follows:

$$\text{ISBL} = \sum_{i=1}^{i=M} C_{e,i,CS} \cdot \left[\left(1 + f_p\right) + \left(f_{er} + f_{el} + f_i + f_C + f_S + f_l\right)\right] \quad (1)$$

$C_{e,I,CS}$ are the equipment cost for the unit based on carbon steel and f_i are the cost factors that can be obtained from Table S1.

Table S1. Values for the factorial method [51].

Type	Factor	Value
Equipment erection	f_{er}	0.3
Piping	f_p	0.8
Instrumentation and control	f_i	0.3
Electrical	f_{el}	0.2
Civil	f_c	0.3
Structures and buildings	f_s	0.2
Lagging and paint	f_l	0.1
Material	f_m	1.0 (carbon steel)

The treatment costs were estimated based on the plant design shown in Figure 9 in the main text, in which the WWTP consists of three tanks, three pumps, one reservoir, one stirrer, and one photoreactor. Even though a tank and a pump were provided for hydrogen peroxide, these two components were neglected for the cost estimate. The total amount of H_2O_2 to be added is very small and the addition can be conducted manually. The standard costs (C_e) for components such as pumps or tanks can be easily obtained from cost correlation curves (Table S2) [51]. The values were calculated from Equation (2) (a stands for the base costs, b and n are values from the individual cost correlation curves, and S is the specific size):

$$C_e = a + b \cdot S^n \quad (2)$$

The quoted prices often refer to a specific year and the United States or United Kingdom as the location. The cost estimation was made for the year 2022, with Germany as the location, and Euro as the currency. To transfer the costs into 2022, the Chemical Plant Cost Index (CEPCI) was used.

Table S2. C_e values (in Euro) for the individual components for Germany in 2022.

Component	#	a	b	n	S	Unit	C_e
Pumps	3	3300	48	1.2	5.0	L/s	EUR 17504
Tanks	3	5700	700	0.7	5.0	m^3	EUR 37887
Reservoir	1	5700	700	0.7	2.5	m^3	EUR 11295
Stirrer	1	4300	0,8	0.8	5.0	kW	EUR 18089

For the photoreactor, no commercial design is available and a photoreactor had to be designed first. For this purpose, a scale-up from the laboratory reactor was performed assuming the same conditions as those in the laboratory reactor. A comparison of the parameters from the laboratory and WWTP reactors is shown in Table S3.

Table S3. Parameters for the laboratory and WWTP reactors.

	Laboratory reactor	WWTP reactor	Factor
Treated Volume (m^3)	0.1	2500	25000
Total Area (m^2)	1.6×10^{-3}	28,05	17531
Irradiation Area (m^2)	1.2×10^{-3}	27	22500
Flow rate (mL/s)	0.2	5000	25000
Linear velocity (m/s)	6.35×10^{-4}	$9{,}8 \times 10^{-2}$	154
# LEDs (365 nm)	16	352941	22059
Mass of catalyst (g)	50×10^{-3}	1170	23400
Loading of catalyst (g/m^2)	43.3	43.3	1
V_{H2O2} (mL)	0.1	2500	25000
C_{Phenol} (ppm)	50	50	1

A scheme of the proposed photoreactor, in which 25 plates (1 m × 1.08 m) provide the required irradiation area, is shown in Figure S6.

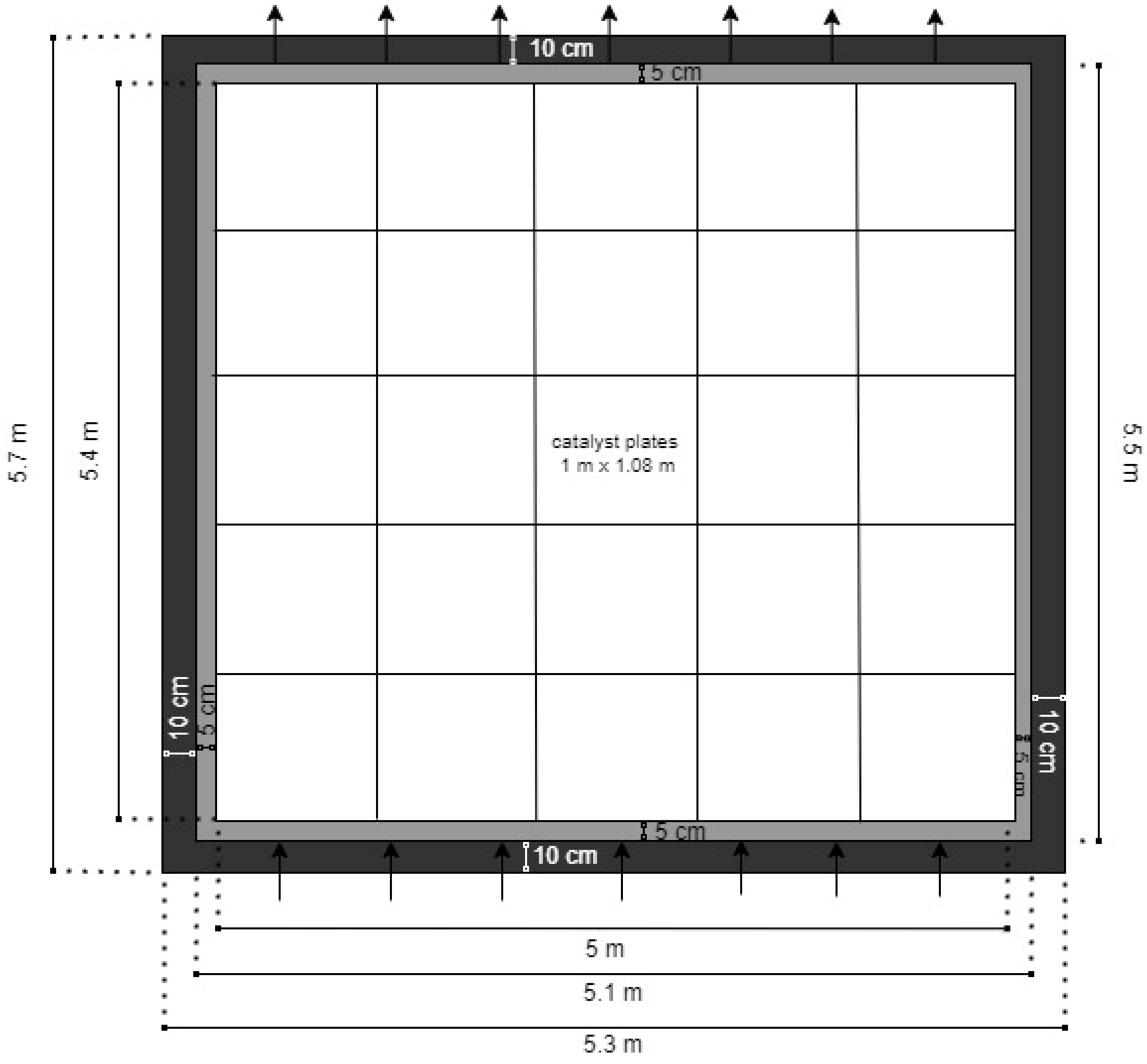

Figure S6. Scheme of the WWTP photoreactor.

The costs for the photoreactor were estimated to be EUR 22855. A steel block (5.7 m × 5.3 m × 1.5 cm) was taken as the body, from which the photoreactor was constructed. The cost for the material was EUR 7618.

This value was multiplied by a factor of three to account for transportation costs, labor, welding, and eventual costs. For the WWTP photoreactor, a window made from quartz glass as used in the laboratory setup was selected. The costs for the required irradiation area were estimated to be EUR 75662. The price for the artificial light source was based on the upscaled amount of LEDs and an individual price of about 3.2 EUR/LED. After having all standard costs, the ISBL (Table S4) was calculated (1) considering the individual factors f_i. Therefore, not all factors were required in every case, e.g., tanks do not need electrical equipment.

Table S4. Individual contributions to ISBL.

Component	C_e	Considered Factors	$1+f_i$	$ISBL_i$
Pumps	EUR 17504	er, p, I, el, c	2.9	EUR 50761
Tanks + Reservoir	EUR 49182	er, p, I, c, l	2.8	EUR 137710
Stirrer	EUR 18089	er, I, c	1.9	EUR 34370
Photoreactor	EUR 22855	er, p, I, c, l	2.8	EUR 63994
Glass window	EUR 75662	er, c	1.6	EUR 121059
			Σ	EUR 407894
Light source	EUR 1129412	er, I, el, c, l		EUR 2484706
			Σ	EUR 2892600

For the WWTP, the ISBL was EUR 2892600 when using an artificial light source and EUR 407894 if sunlight was used. Based on the calculated ISBL, Sinnot and Towler [51] as well as Buchner et al. [56] showed the factors that allow for the calculation of OSBL. Then, ISBL and OSBL were added up to obtain the FCI. In the subsequent step, CapEx was calculated using the FCI, the contingency and the working capital/start-up expenses. Contingency and working capital was, again, obtained using grossing factors based on the FCI. The calculation for the WWTP plant is shown in Table S5.

Table S5. Calculation of CapEx.

Contribution	Artificial Light	Sunlight
ISBL	EUR 2892600	EUR 407894
OSBL (=0.4·ISBL)	EUR 1157040	EUR 163158
FCI (=ISBL+OSBL)	EUR 4049640	EUR 571052
Contigency (=0.1·FCI)	EUR 404964	EUR 57105
Working Capital/start up expanses (=0.05·FCI)	EUR 202482	EUR 28553
CapEx (=FCI+Con+WC)	EUR 4657086	EUR 656710

The operational expenditure (OpEx, Table S6) for the WWTP consists of the electricity costs for the equipment and the costs for hydrogen peroxide and the catalyst, including the chemicals to produce a stable photocatalyst layer. The timescale was set to one year. The electricity costs were calculated for both cases with and without artificial light source. Two batches of contaminated water were assumed to run per day.

Table S6. Calculation of OpEx.

Costs	Artificial Light	Sunlight
electricity	EUR 190193	EUR 2754
chemicals	EUR 497	EUR 497
catalyst	EUR 878	EUR 878
Σ =	EUR 191570	EUR 4130

The total treatment costs were calculated by adding the operational expenditure per year to the annual depreciation expenses. The depreciation was planned to be 10 years so the CapEx had to be divided by a factor of 10. Then, the sum of OpEx per year and annual depreciation expense was divided by the amount of treated wastewater per year to obtain the cost for one cubic meter of treated wastewater. The treatment costs for both cases are shown in Table S7 for 1150 m^3 of wastewater to be treated in total.

Table S7. Calculation of the total treatment costs.

Costs	Artificial Light	Sunlight
Annual depreciation expense (=0.1·CapEx)	EUR 465709	EUR 65671
OpEx	EUR 191570	EUR 4130
Total cost per year	EUR 657279	EUR 69801
Treated volume per year	1150 m^3	1150 m^3
Treatment cost per m^3	572 EUR/m^3	61 EUR/m^3

The WWTP reactor was designed to have the operating and material characteristics of the laboratory reactor. Regardless of whether the irradiation is applied with sunlight or an artificial light source, a cost saving can be achieved by changing the materials, e.g., using Teflon instead of stainless steel for the housing, since only aqueous solutions have to be treated, and a UV transmissive polymer, e.g., Plexiglas, instead of quartz glass. Including the cost savings for the photoreactor, the treatment costs for sunlight irradiation could be about 40 EUR/m^3, which is still high.
